# Indole-3-carboxamide alleviates LPS-induced endometritis through suppressing ferroptosis and inflammation via regulating Aryl hydrocarbon receptor

**DOI:** 10.3389/fcimb.2025.1649801

**Published:** 2025-10-09

**Authors:** Feng Chen, Rujin Cheng, Xiangyang Chen, Zhiheng Guo

**Affiliations:** Gynaecology and Obstetrics Center, The First Hospital of Jilin University, Changchun, Jilin, China

**Keywords:** endometritis, LPS, AhR, indole-3-carboxamide, ferroptosis

## Abstract

**Purpose:**

Indole-3-carboxamide (I3A), a derivative of tryptophan indole, has been reported to have anti-inflammatory role. This study aims to investigate the effects of I3A on LPS-induced endometritis in mice.

**Methods:**

The mice endometritis model was established and I3A was administered to mice orally (150 mg/kg/day) for two days. TNF-α and IL-1β production were analyzed by ELISA. The protein expression was measured by western blot. The pathological changes of mouse uterine tissue were observed by H&E staining.

**Results:**

The experimental results showed I3A significantly alleviated LPS-induced uterine pathological injury. I3A treatment obviously attenuated LPS-induced MPO activity, TNF-α and IL-1β production. I3A also inhibited LPS-induced ferroptosis and NF-κB activation. Furthermore, I3A could up-regulate the expression of AhR and SLC7A11. The protective role of I3A on LPS-induced endometritis was reversed by AhR inhibitor CH223191.

**Conclusion:**

In conclusion, I3A inhibits LPS-induced endometritis in mice by attenuating inflammation and ferroptosis via the AhR-SLC7A11 signaling pathway.

## 1 Introduction

Chronic endometritis is an inflammatory response that occurs when the endometrium is infected with pathogens (Kimura et al., 2019). Women of childbearing age may not only experience symptoms such as abdominal pain, fever, and menstrual abnormalities, but may also suffer from infertility or habitual miscarriage (Negishi et al., 2021). When inflammation occurs in the endometrium, immune cells and inflammatory factors can interfere with the signal communication between the embryo and the endometrium, thereby affecting embryo implantation (van Mourik et al., 2009). Antibiotic management is still the main treatment method for endometritis (Sfakianoudis et al., 2018). Although antibiotics can cure some endometritis and improve the pregnancy outcomes of patients, there are still some patients with persistent endometrial inflammation after regular antibiotic management in clinical treatment (Cicinelli et al., 2018). Therefore, avoiding the abuse of antibiotics and seeking effective treatment methods to improve fertility for endometritis are urgent issues that need to be addressed.

Ferroptosis, as a form of programmed cell death driven by iron dependent lipid peroxidation, is characterized by pathological changes including inhibition of GPX4 activity, accumulation of lipid free radicals, and disappearance of mitochondrial cristae (Zhou et al., 2025). In recent years, ferroptosis has been shown to play an important role in endometritis (Cao et al., 2023). A previous study showed that ferroptosis was involved in the development of endometritis. Inhibition of ferroptosis could attenuate LPS or S.aureus-induced endometritis (Zhao et al., 2023; Yu et al., 2024).

Indole-3-carboxamide (I3A), a derivative of tryptophan indole, is a unique metabolite of microorganisms and has been shown to have certain anti-inflammatory and anti-tumor activities (Huang et al., 2023). A previous study showed that I3A exhibited radioprotective effect against intestinal organoids (Xie et al., 2024). I3A has been known to inhibit liver injury induced by acetaminophen through alleviating oxidative stress (Liu et al., 2024). Furthermore, I3A could attenuate kidney injury induced by cisplatin via suppressing apoptosis and increasing mitochondrial function (Yuan et al., 2024). I3A could alleviate aortic dissection through inhibiting inflammatory response (Huang et al., 2023). In addition, I3A has been reported to inhibit DSS-induced colitis through attenuating inflammation and increasing gut barrier function (Liu et al., 2023). However, the therapeutic effect of I3A on endometritis has not been reported. Therefore, we constructed a mouse endometritis model to investigate the therapeutic effect and mechanism of I3A on endometritis.

## 2 Materials and methods

### 2.1 Reagents

LPS and I3A were obtained from Sigma–Aldrich (MO, USA). The ELISA kits for mouse TNF-α, and IL-1β were purchased from Biolegend company (CA, USA). The BCA protein quantification kit and T-PER tissue protein lysate were all purchased from Thermo Scientific. Goat anti rabbit HRP conjugated IgG secondary antibody was purchased from Tianjin Sanjian Biotechnology Co., Ltd. AhR (#83200), ZO-1 (#13663), occludin (#91131), GPX4 (#52455), SLC7A11 (#98051), p65 (#6956), p-p65 (#3033), p-IκBα (#2859), IκBα (#9242), and β-actin (#4967) primary antibodies were purchased from CST company.

### 2.2 Experimental design and grouping

C57BL/6J mice were purchased from the SPF Experimental Animal Center of Jilin University, using 8-week-old female C57BL/6J mice with a body weight of 20-25g. Mice were housed in independent ventilated cages, with free access to food and water. The experimental period began after one week of adaptive feeding. The mouse endometritis model was established as previous described (Wang et al., 2022). Sixty female C57BL/6J mice were randomly divided into five groups and each group contained twelve mice: control group, I3A group, LPS group, LPS + I3A group, and LPS + I3A + CH223191 group. I3A was administered to mice orally (150 mg/kg/day) for two days. CH223191 was given via oral gavage (20mg/kg/day) for two days. 24 h after LPS treatment, the uterine tissue was collected for subsequent experiments. This research protocol has been approved by the Institutional Animal Ethics Committee of Jilin University.

### 2.3 H&E staining

Mouse uterine tissue was fixed in a 10% formaldehyde solution for 48 hours and embedded in paraffin. It was sliced into 4 μm thick sections using a microtome, dehydrated with different concentrations of alcohol (100%, 75%, 50%, and 25%), and finally stained with hematoxylin and eosin (H&E). Uterine histopathological changes were observed through an optical microscope.

### 2.4 Ferroptosis biomarkers detection

The levels of ferroptosis biomarkers Fe^2+^, malondialdehyde (MDA), glutathione (GSH), MPO, and glutathione peroxidase 4 (GPX4) activity in mouse uterine tissue were measured using iron assay kit, MDA analysis kit, GSH analysis kit, and GPX4 protein expression according to the instructions.

### 2.5 Inflammatory cytokine assay

The production of TNF-α and IL-1β in uterine tissues were tested by the ELISA kits according to the manufacturer’s protocol.

### 2.6 Western blot analysis

The protein was extracted using the kit and protein concentration was detected by the BCA method. After protein denaturation, 10% SDS-PAGE was performed, followed by semi dry membrane transfer. The membrane was then blocked with 3% BSA for 4 hours and incubated overnight at 4 °C with primary antibodies including GPX4, p-p65, p-p38, AhR, ZO-1, and occludin (diluted at 1:1000). The membrane was washed with TBST and incubated at room temperature with goat anti rabbit HRP conjugated IgG secondary antibody (diluted at 1:5000) for 1 hour. The PVDF membrane was washed again with TBST, developed with a chemiluminescence substrate, and images were collected using a chemiluminescence imaging system. The grayscale values of the results were analyzed using ImageJ software.

### 2.7 *In vitro* experiment

The mouse uterine epithelial cells (MUECs) were isolated from uterine tissues as previously described. The experiment was divided into five groups: control group, I3A group, LPS group, LPS + I3A group, and LPS + I3A + CH223191 group. The cells were treated with I3A (50 μM) and then stimulated by LPS (10 μg/mL). For AhR inhibitory experiment, the cells were treated with CH223191 (20 μM) and then treated with I3A (50 μM) and then stimulated by LPS (10 μg/mL). Finally, the cells and the cell supernatants were collected. The effects of I3A on LPS-induced inflammation and ferroptosis were detected.

### 2.8 Statistical analysis

All statistical analyses were conducted using GraphPadPrism8.0 statistical software. The data is presented as the mean ± standard deviation of at least three independent experiments. Two independent samples t-test was used for comparison between two groups. For multi group comparison, one-way ANOVA was first used to compare the differences in the mean values of each group, and then Tukey’s multiple comparison analysis was used to compare the differences between pairs. P<0.05 indicates statistical significance.

## 3 Results

### 3.1 I3A attenuates LPS-induced uterine injury

**Figure 1:**
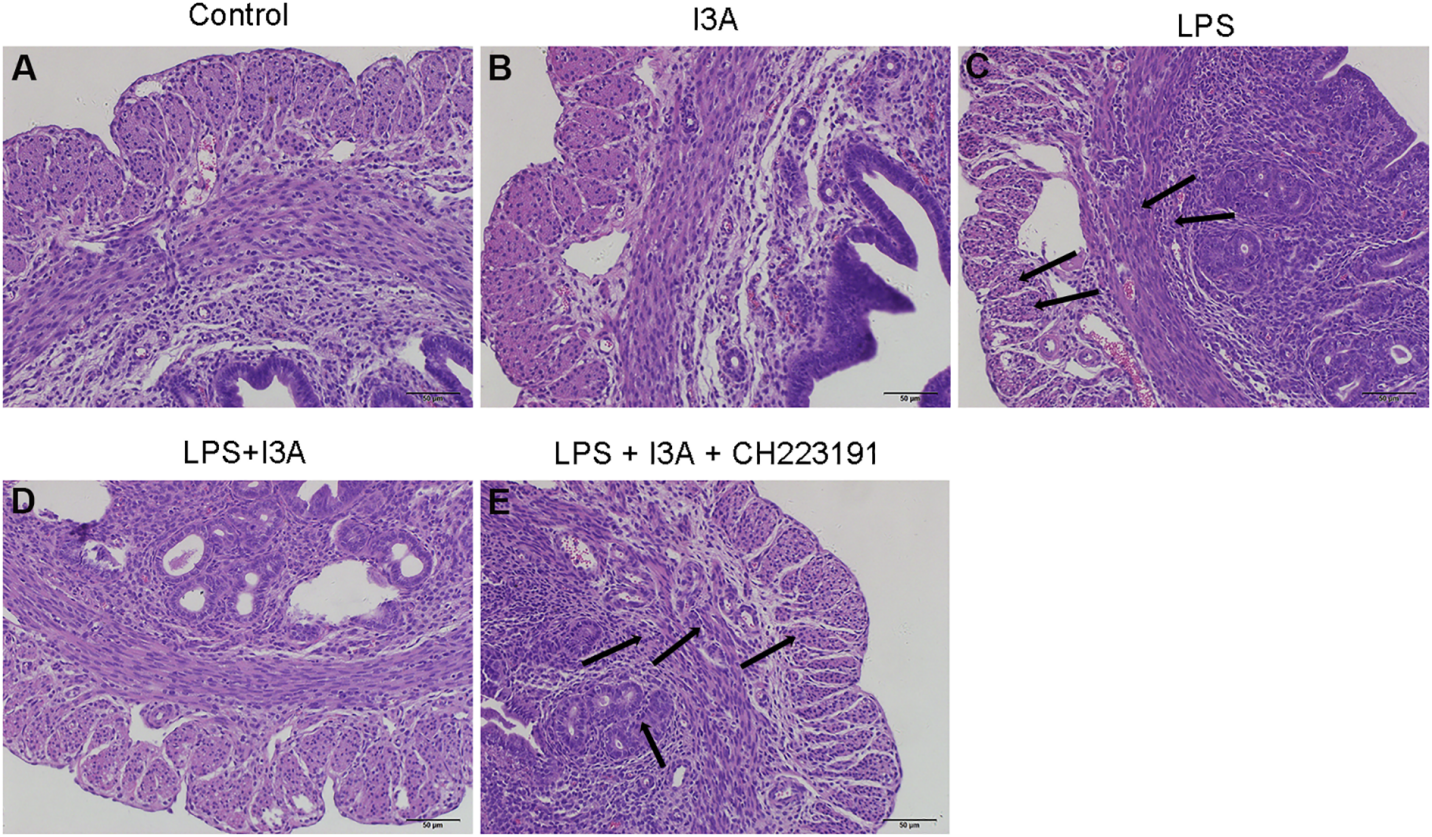
Effects of I3A on LPS-induced uterine histopathological changes. Histopathologic sections of uterine tissues (H&E, × 100). **(A)** control group, **(B)** I3A group, **(C)** LPS group, **(D)** LPS + I3A group, **(E)** LPS + I3A + CH223191 group.

Pathological sections of mouse uterus showed normal tissue in the control group and I3A group, while significant neutrophil infiltration, epithelial hyperplasia, edema, necrosis, and shedding were observed in the LPS group, indicating the successful construction of the mouse endometritis model (**Figures 1A–C**). After I3A treatment, the uterine tissue basically returned to normal (**Figure 1D**). The uterine tissue of the LPS + I3A + CH223191 group showed significantly pathological damage (**Figure 1E**).

### 3.2 I3A attenuates LPS-induced MPO activity

**Figure 2:**
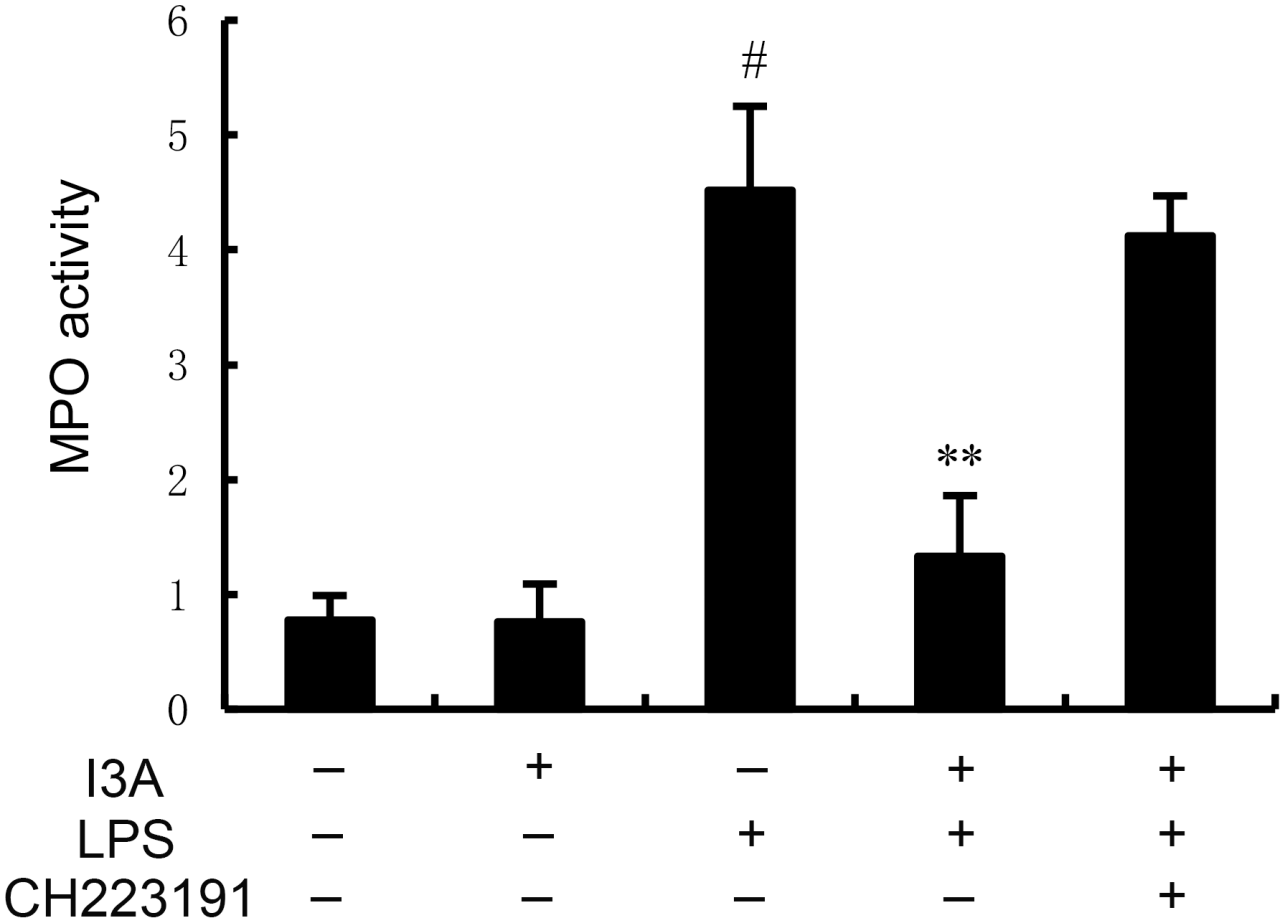
Effect of I3A on MPO activity. The values presented are the mean ± SD. ^#^ *P* < 0.01 is significantly different from control group; ^**^ *P* < 0.01 are significantly different from LPS group.

The MPO activity of I3A group was nearly the same with the control group. Compared with the control group, the MPO activity in the uterine tissue of the LPS group significantly increased (P<0.01). Compared with LPS group, the MPO activity of LPS+I3A group significantly decreased (P<0.01). However, the inhibition of I3A on LPS-induced MPO activity was reversed by AhR inhibitor CH223191 (**Figure 2**).

### 3.3 I3A attenuates LPS-induced TNF-α and IL-1β production

**Figure 3:**
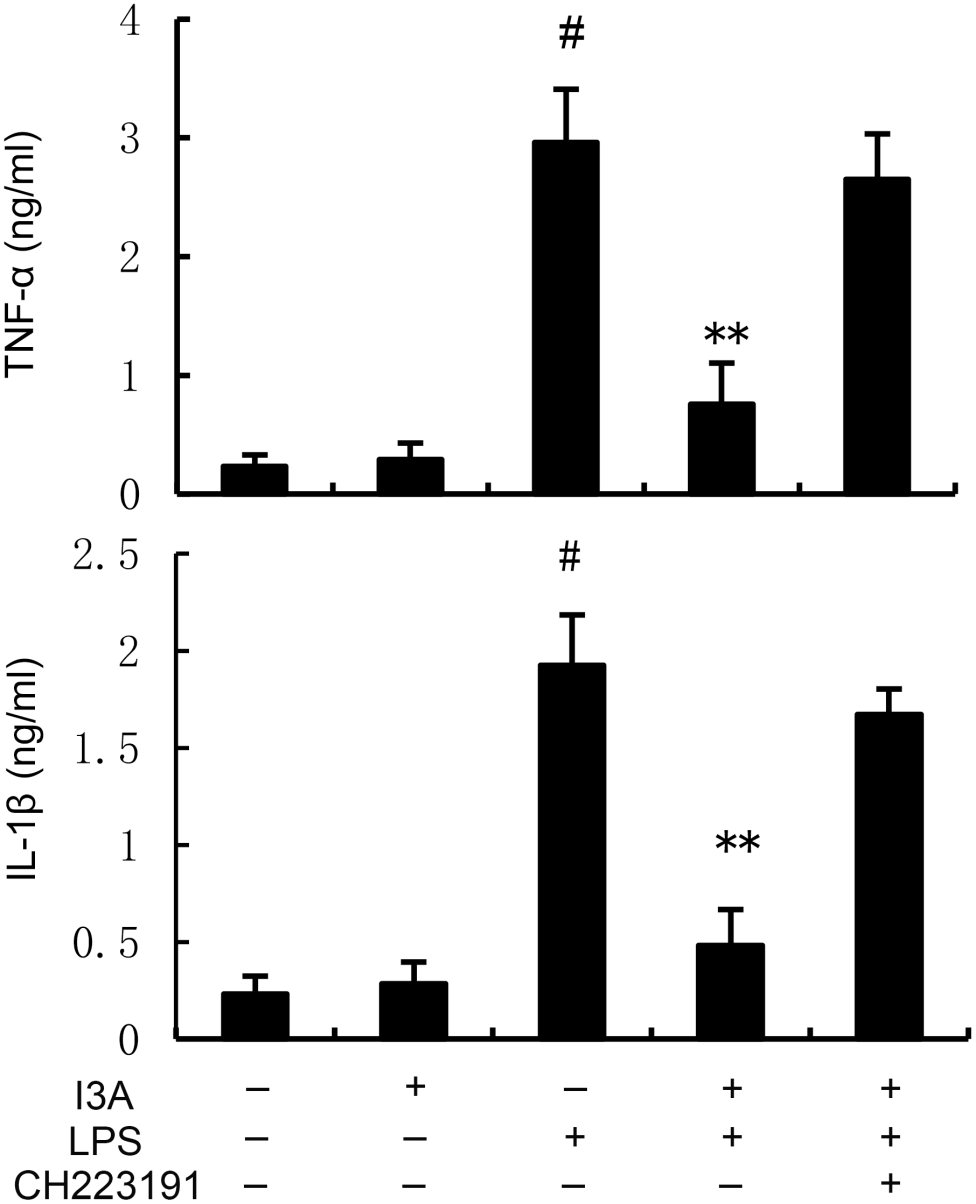
Effect of I3A on inflammatory cytokine production. The values presented are the mean ± SD. ^#^ *P* < 0.01 is significantly different from control group; ^**^ *P* < 0.01 are significantly different from LPS group.

The TNF-α and IL-1β levels of I3A group was nearly the same with the control group. Compared with the control group, the TNF-α and IL-1β levels in the uterine tissue of the LPS group significantly increased (P<0.01). Compared with LPS group, the TNF-α and IL-1β levels of LPS+I3A group significantly decreased (P<0.01). However, the inhibition of I3A on LPS-induced TNF-α and IL-1β levels was reversed by AhR inhibitor CH223191 (**Figure 3**).

### 3.4 I3A inhibits LPS-induced ferroptosis

**Figure 4:**
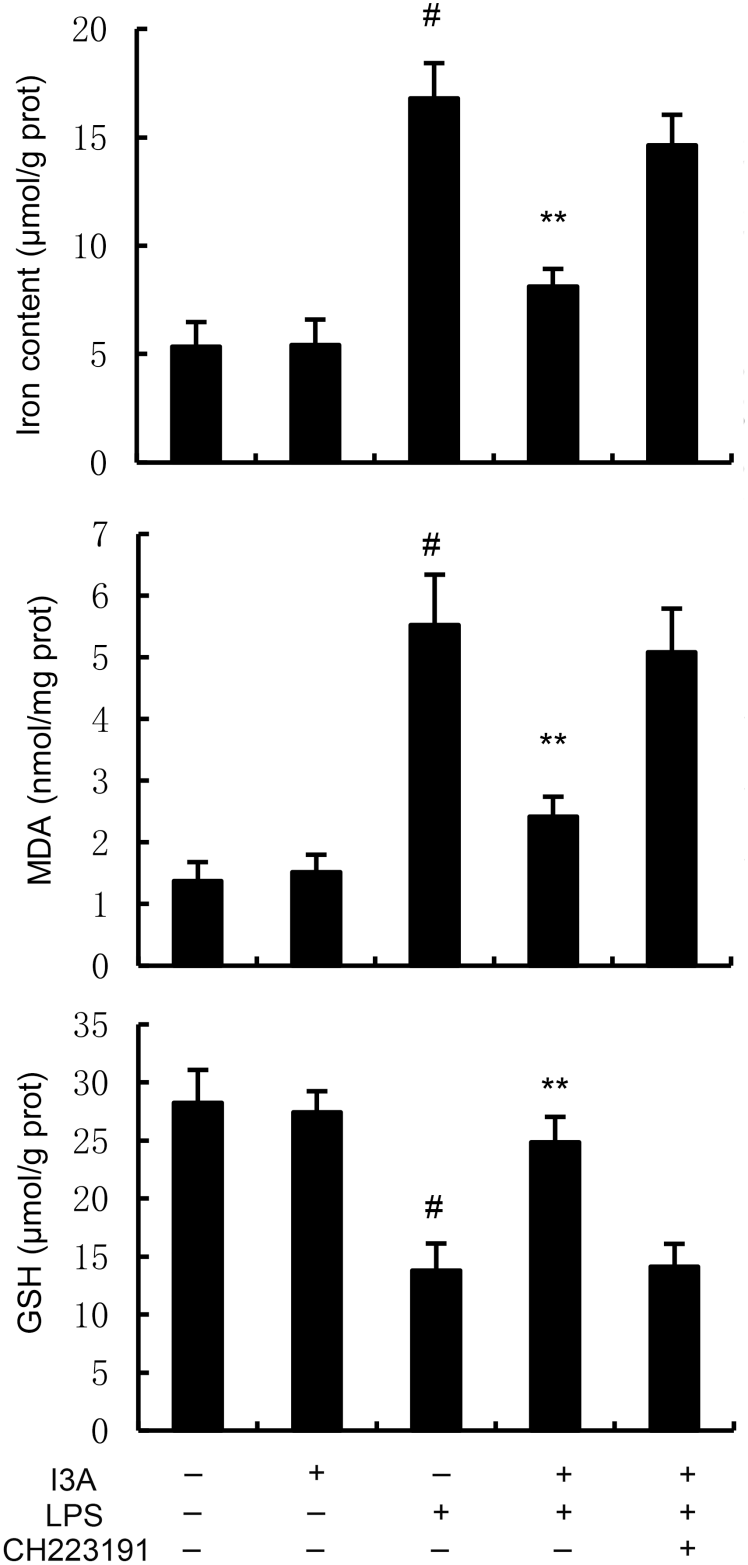
Effect of I3A on MDA, iron, and GSH production. The values presented are the mean ± SD. ^#^ *P* < 0.01 is significantly different from control group; ^**^ *P* < 0.01 are significantly different from LPS group.

**Figure 5:**
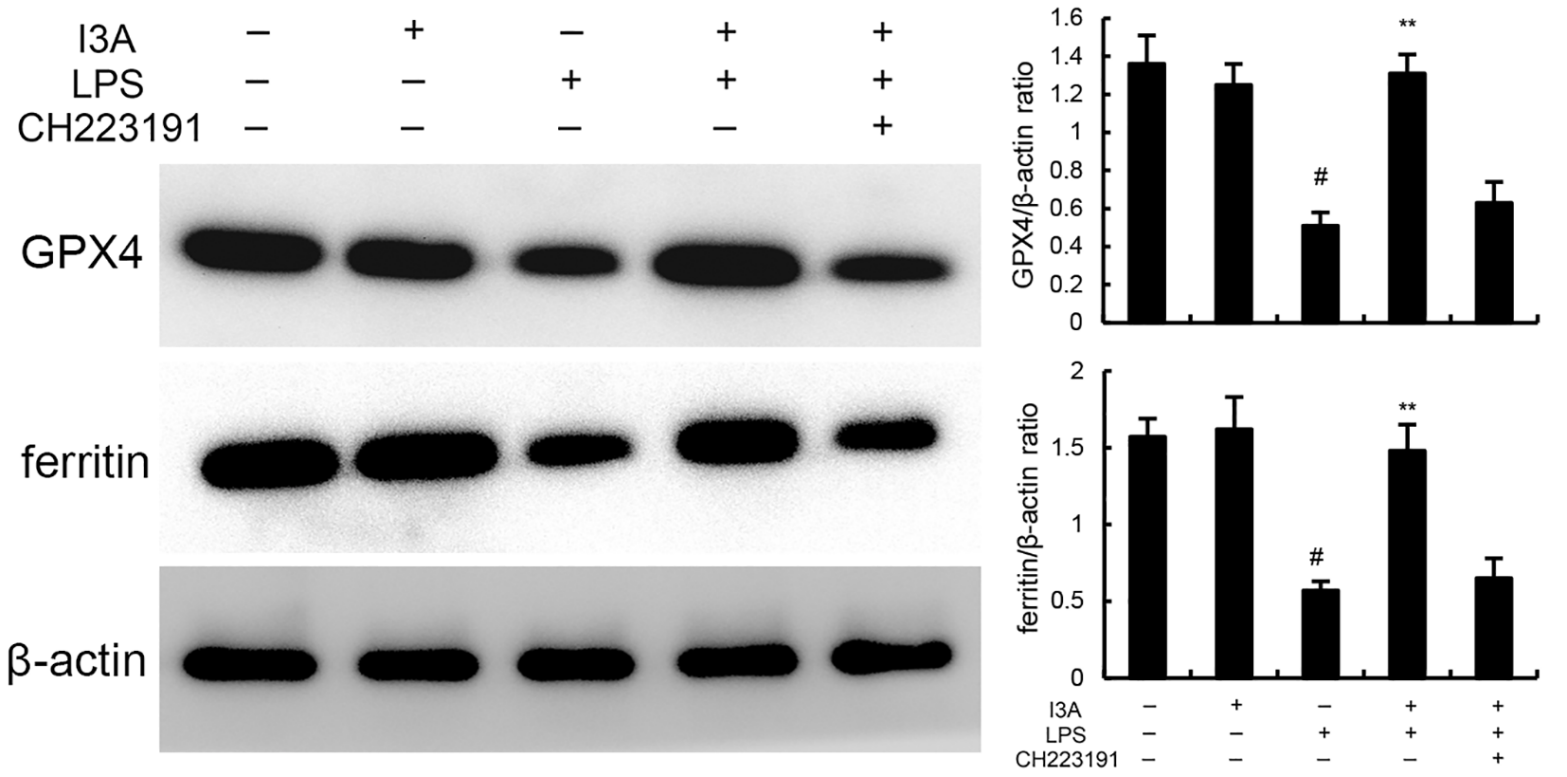
Effect of I3A on GPX4 and ferritin expression. The values presented are the mean ± SD. ^#^ *P* < 0.01 is significantly different from control group; ^**^ *P* < 0.01 are significantly different from LPS group.

The levels of MDA, iron, and GSH of I3A group were nearly the same with the control group. Compared with the control group, the levels of MDA and iron in the uterine tissue of the LPS group significantly increased (P<0.01). Compared with LPS group, the levels of MDA and iron of LPS+I3A group significantly decreased (P<0.01). However, the inhibition of I3A on LPS-induced MDA and iron production was reversed by AhR inhibitor CH223191 (**Figure 4**). Meanwhile, compared with the control group, the level of GSH and GPX4 expression in the uterine tissue of the LPS group significantly decreased (P<0.01). Compared with LPS group, the level of GSH and GPX4 and ferritin expression of LPS+I3A group significantly increased (P<0.01). However, the up-regulation of I3A on the level of GSH and GPX4 expression were reversed by AhR inhibitor CH223191 (**Figure 5**).

### 3.5 I3A inhibits LPS-induced NF-κB activation

**Figure 6:**
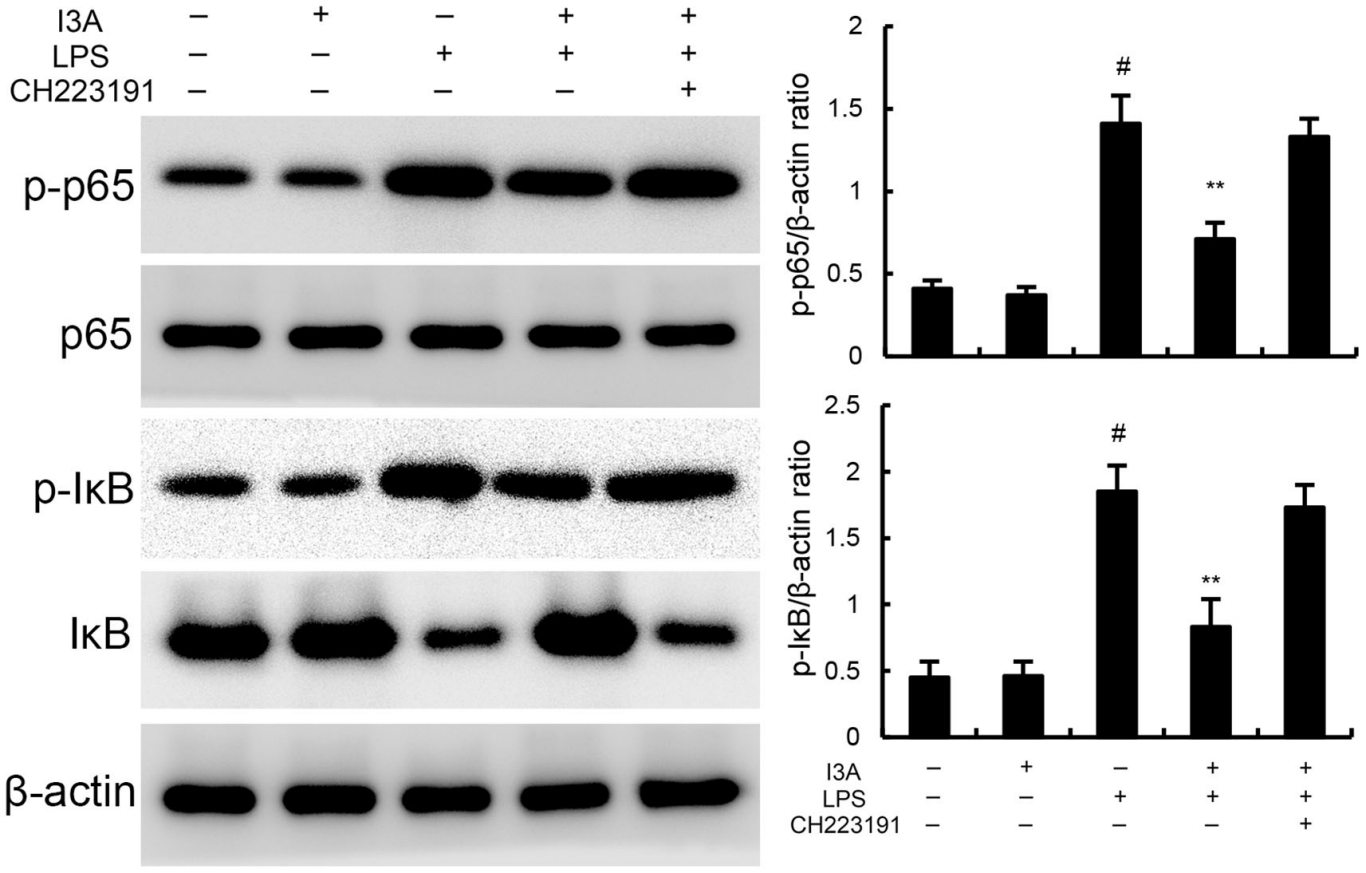
Effect of I3A on NF-κB activation in uterine tissues. The values presented are the mean ± SD. ^#^ *P* < 0.01 is significantly different from control group; ^**^ *P* < 0.01 are significantly different from LPS group.

The levels of NF-κB p-p65 and p-IκBα of I3A group was nearly the same with the control group. Compared with the control group, the levels of NF-κB p-p65 and p-IκBα in the uterine tissue of the LPS group significantly increased (P<0.01). Compared with LPS group, the levels of NF-κB p-p65 and p-IκBα of LPS+I3A group significantly decreased (P<0.01). However, the inhibition of I3A on LPS-induced NF-κB p-p65 and p-IκBα levels was reversed by AhR inhibitor CH223191 (**Figure 6**).

### 3.6 Effects of I3A on AhR expression

**Figure 7:**
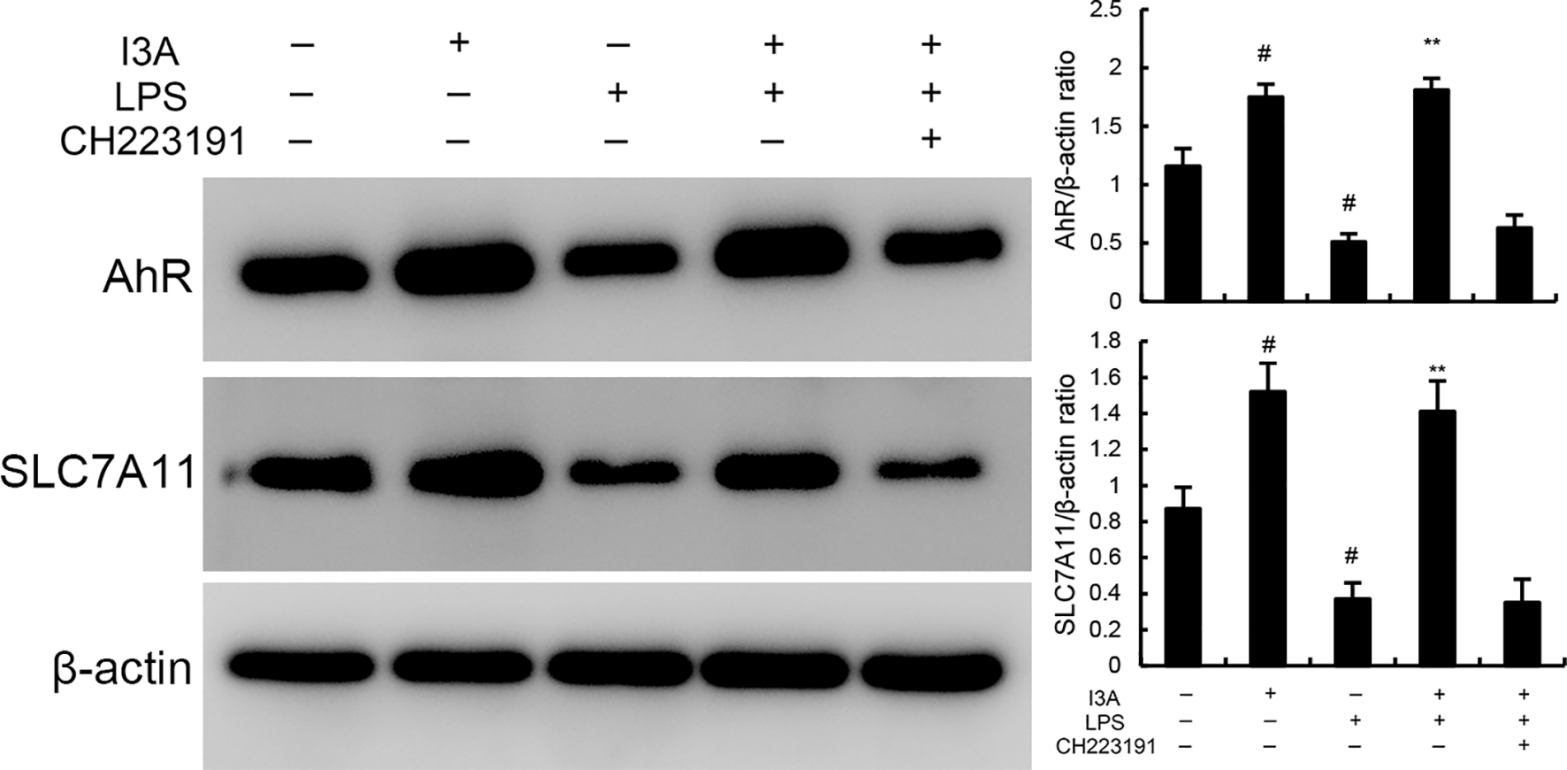
Effects of I3A on AhR/SCL7A11 signaling pathway. The values presented are the mean ± SD. ^#^ *P* < 0.01 is significantly different from control group; ^**^ *P* < 0.01 are significantly different from LPS group.

The expression of AhR and SLC7A11 of I3A group was higher than the control group. Compared with the control group, the expression of AhR and SLC7A11 in the uterine tissue of the LPS group significantly decreased (P<0.01). Compared with LPS group, the expression of AhR and SLC7A11 of LPS+I3A group significantly increased (P<0.01). However, the up-regulation of I3A on LPS-induced AhR and SLC7A11 expression was reversed by AhR inhibitor CH223191 (**Figure 7**).

### 3.7 I3A attenuates LPS-induced inflammation and ferroptosis through regulating AhR *in vitro*

**Figure 8:**
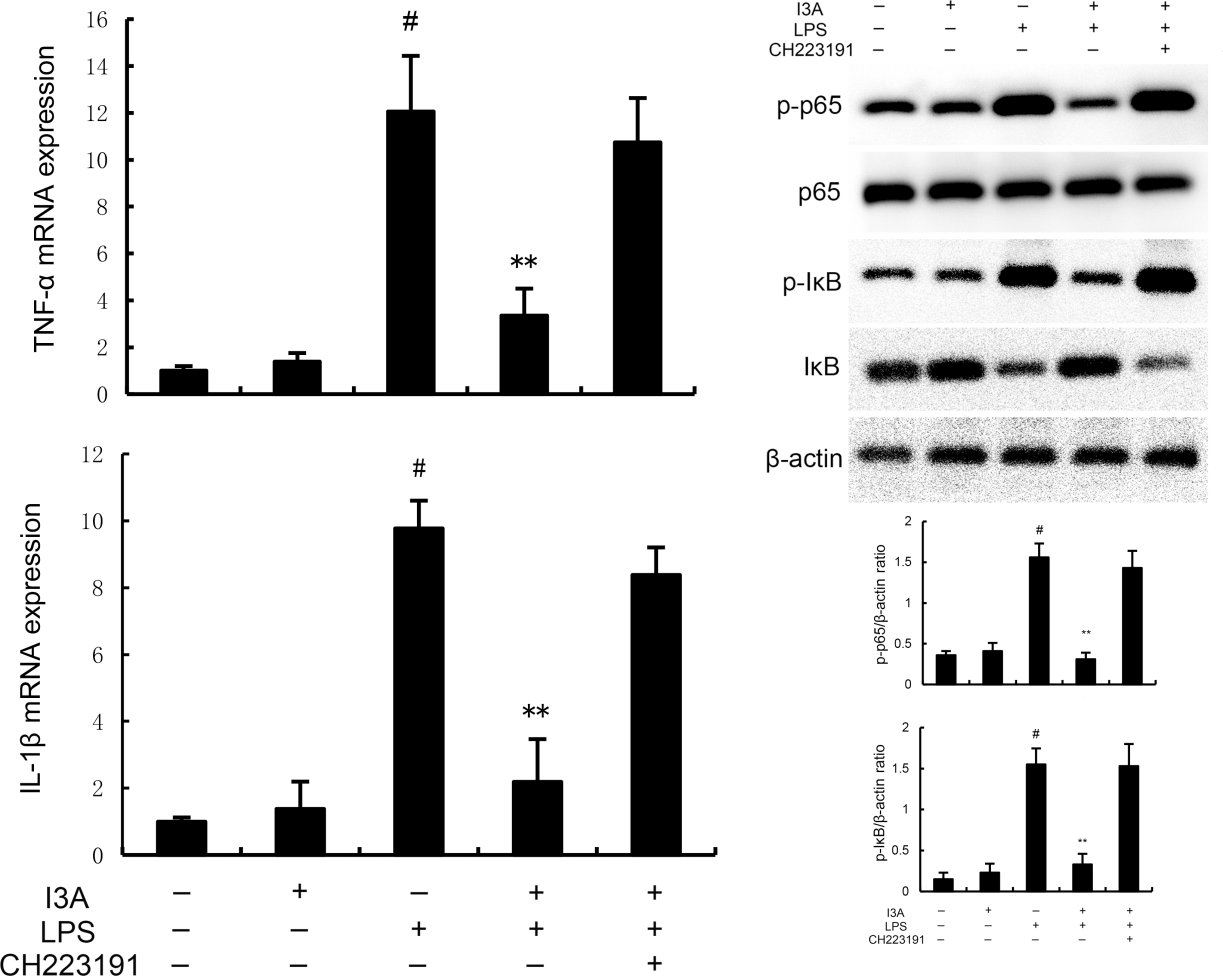
I3A inhibits LPS-induced inflammation and NF-κB activation *in vitro*. The values presented are the mean ± SD. ^#^P < 0.01 is significantly different from control group; **P < 0.01 are significantly different from LPS group.

**Figure 9:**
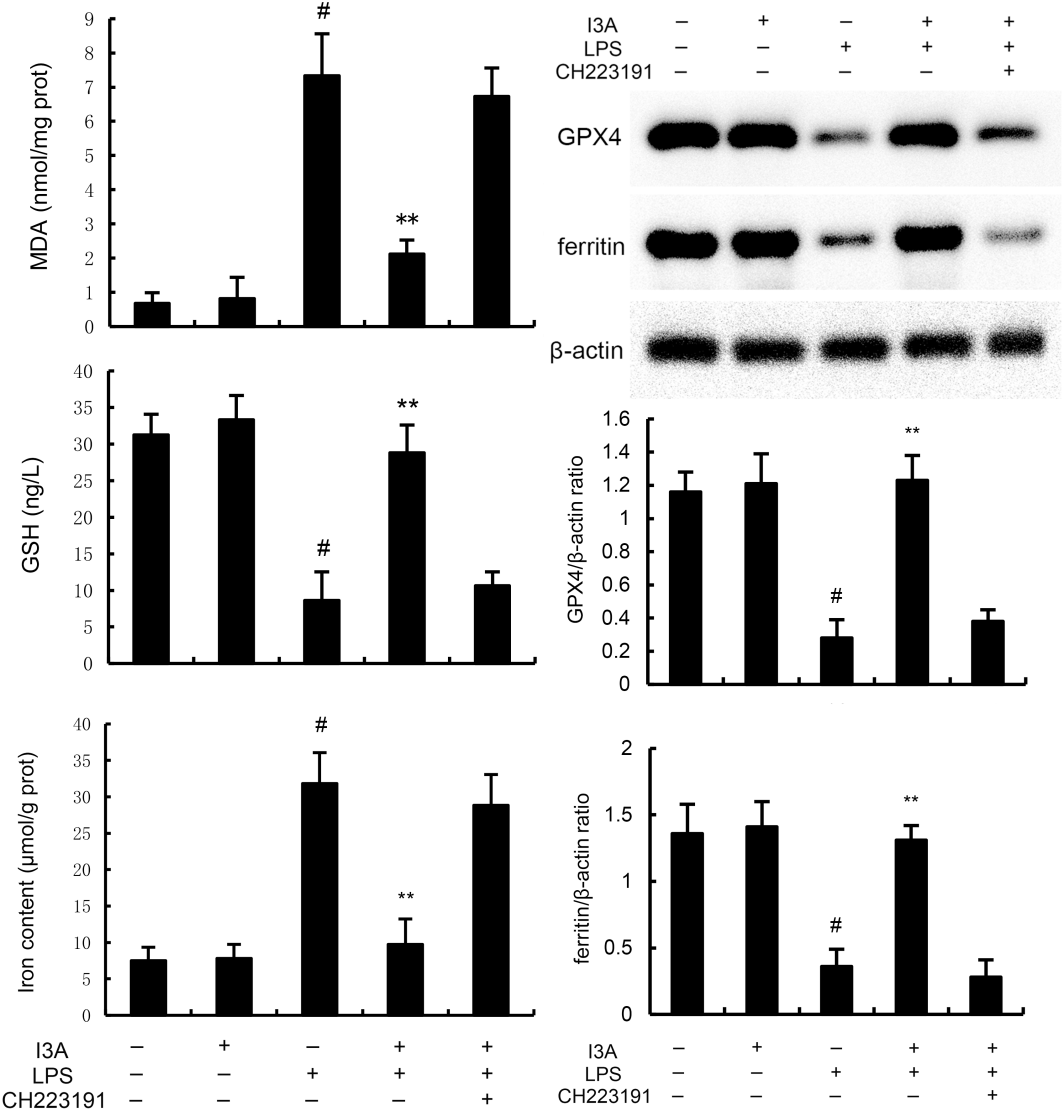
I3A inhibits LPS-induced ferroptosis *in vitro*. The values presented are the mean ± SD. ^#^P < 0.01 is significantly different from control group; **P < 0.01 are significantly different from LPS group.

**Figure 10:**
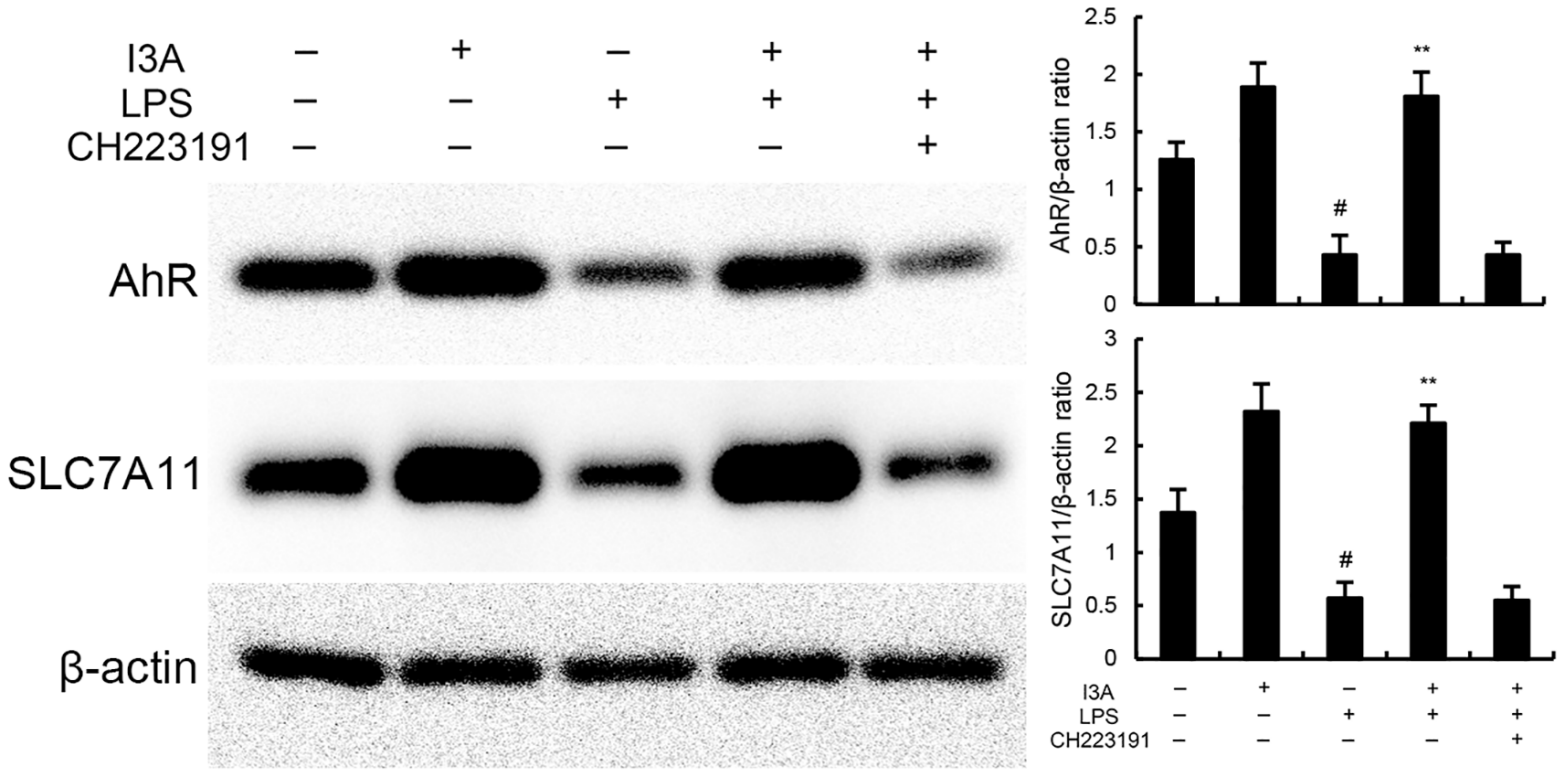
Effects of I3A on AhR/SCL7A11 signaling pathway *in vitro*. The values presented are the mean ± SD. ^#^ *P* < 0.01 is significantly different from control group; ^**^ *P* < 0.01 are significantly different from LPS group.

As demonstrated in **Figure 8**, LPS stimulation significantly increased the mRNA expression of TNF-α and IL-1β. Treatment of I3A attenuated the expression of TNF-α and IL-1β, and NF-κB activation induced by LPS. However, the inhibition was prevented by AhR inhibitor CH223191 (**Figure 8**). LPS stimulation markedly increased the production of MDA and Fe^2+^ and decreased GSH level, GPX4 and ferritin expression. However, these changes were reversed by AhR inhibitor CH223191 (**Figure 9**). Meanwhile, LPS stimulation decreased the expression of AhR and SLC7A11. Treatment of I3A significantly up-regulated the expression of AhR and SLC7A11. However, the up-regulation of I3A on LPS-induced AhR and SLC7A11 expression was reversed by AhR inhibitor CH223191 (**Figure 10**).

## 4 Discussion

Endometritis is a type of female pelvic inflammatory disease and a common factor leading to adverse pregnancy outcomes in women of childbearing age (Ravel et al., 2021). The pathological cause of infertility may be the persistent inflammatory response of the endometrium after infection, leading to the accumulation of a large number of plasma cells and lymphocytes in the endometrium, changing the immune environment of the endometrium, resulting in spermicidal and embryotoxic effects (Kitaya et al., 2016). At the same time, the receptivity of the endometrium is also adversely affected, leading to difficulties in embryo implantation and conception (Drizi et al., 2020). In the later stage of assisted reproduction, there may also be repeated failures in embryo implantation. In this study, we found that I3A significantly inhibited LPS-induced endometritis through inhibition inflammation and ferroptosis.

Gram-negative bacterium *Escherichia coli* (*E. coli*) has been identified as the main pathogenic microorganism causing endometritis (Jana et al., 2007). LPS is the main component of the cell wall of *Gram-negative* bacteria and one of the most effective stimulators of the immune system (Matsuura, 2013). When it enters the body, it can activate the NF-κB pathway and release inflammatory cytokines such as TNF-α and IL-1β, leading to inflammation and damage to the body (Tang et al., 2021). In this study, LPS stimulation significantly increased the phosphorylation levels of NF-κB p65 and IκB, while I3A treatment significantly inhibited the phosphorylation levels of NF-κB p65 and IκB. Meanwhile, I3A significantly inhibited LPS-induced inflammatory response. Previous studies demonstrated that indole metabolites, such as indole-3-carbinol (I3C), indole-3-acetic acid (IAA), indole-3-propionic acid (IPA), exhibited anti-inflammatory effects (Li et al., 2025; Liu et al., 2025). IPA could inhibit LPS-induced pro-inflammatory cytokine production (TNF-α, IL-6) in mouse macrophages by suppressing NF-κB phosphorylation (Huang et al., 2022). IAA could alleviate DSS-induced colitis through inhibiting inflammation and NF-κB activation (Li et al., 2024). These studies collectively confirm that indole derivatives share a conserved anti-inflammatory mechanism against LPS-driven tissue injury, supporting the validity of I3A’s role in endometritis. Ferroptosis is a new type of programmed cell death that is different from apoptosis and cell necrosis (Yu et al., 2017). Its mechanism involves iron overload caused by abnormal iron metabolism, lipid peroxidation, and reactive oxygen species generation (Latunde-Dada, 2017). Recent studies showed that ferroptosis was related to the development of endometritis (Cao et al., 2023). And inhibition of ferroptosis could protect mice against endometritis (Wang et al., 2022). In this study, I3A significantly attenuated LPS-induced ferroptosis.

Aryl hydrocarbon receptor (AhR) is a ligand activated transcription factor that regulates the body’s response to environmental stimuli (Rothhammer and Quintana, 2019). AhR has been increasingly recognized as an important disease regulatory factor in recent years, particularly in regulating immune and inflammatory responses (Neavin et al., 2018). AhR is also a highly diverse nuclear receptor that can bind to a variety of different ligands. These ligands include exogenous synthesized aromatic hydrocarbons, exogenous natural chemicals, and endogenous ligands (Abel and Haarmann-Stemmann, 2010). Specifically, the tryptophan (TRP) pathway provides many ligands for AhR and plays important roles in immune and inflammatory responses (Sun et al., 2020). I3A, a derivative of tryptophan indole, has been known to exhibit anti-inflammatory role. In this study, I3A significantly increased the expression of AhR. SLC7A11 is one of the important components of the Xc- antiporter protein (Jyotsana et al., 2022). The Xc- system is a non-sodium dependent antiporter that outputs intracellular glutamate and inputs extracellular cysteine in a 1:1 ratio. SLC7A11 is a multi-channel transmembrane protein that mediates the activity of cystine and glutamate antiporters in the Xc- system (Wang et al., 2023). Recently, many studies have reported that various SLC7A11 regulatory factors regulate the sensitivity of cells to ferroptosis by modulating the expression or activity of SLC7A11 (Feng et al., 2021). SLC7A11 can inhibit ferroptosis and promote GSH biosynthesis by inputting cysteine, thereby promoting GPX4 mediated lipid peroxidation process (Hu et al., 2022). AhR modulates ferroptosis primarily by directly regulating SLC7A11 transcription (Kou et al., 2024). In many physiological or adaptive contexts, activated AhR promotes SLC7A11 expression to reinforce redox defense and suppress ferroptosis (Fiore et al., 2022; Peng et al., 2023). The AhR-SLC7A11-ferroptosis axis has critical relevance to human disease and therapy development. Therefore, we detected SLC7A11 in this study. In this study, I3A significantly up-regulated the expression of SLC7A11, suggesting I3A inhibited ferroptosis might through AhR-SLC7A11 signaling pathway.

In summary, I3A can significantly alleviate the symptoms of endometritis in mice. Its mechanism is to activate AhR and inhibit the NF-κB signaling pathway, which leads to the inhibition of inflammatory cytokines and alleviates endometritis, thereby achieving therapeutic effects. In further research, we will conduct long-term oral toxicity studies in mice or rats to assess I3A’s effects on reproductive function. It is critical for its use in reproductive-age women or breeding livestock.

## Data Availability

The original contributions presented in the study are included in the article/supplementary material. Further inquiries can be directed to the corresponding author.
